# Interferon-Beta Increases Plasma Ceramides of Specific Chain Length in Multiple Sclerosis Patients, Unlike Fingolimod or Natalizumab

**DOI:** 10.3389/fphar.2016.00412

**Published:** 2016-11-03

**Authors:** Florian M. Ottenlinger, Christoph A. Mayer, Nerea Ferreirós, Yannick Schreiber, Anja Schwiebs, Katrin G. Schmidt, Hanns Ackermann, Josef M. Pfeilschifter, Heinfried H. Radeke

**Affiliations:** ^1^Pharmazentrum Frankfurt, Institute of Pharmacology and Toxicology, Goethe University FrankfurtFrankfurt am Main, Germany; ^2^Center for Neurology and Neurosurgery, Goethe University FrankfurtFrankfurt am Main, Germany; ^3^Pharmazentrum Frankfurt/ZAFES, Institute for Clinical Pharmacology, Goethe University FrankfurtFrankfurt am Main, Germany; ^4^Fraunhofer Institute for Molecular Biology and Applied Ecology IME, Project Group TMP, Goethe University FrankfurtFrankfurt am Main, Germany; ^5^Institute for Biostatistics and Mathematical Modelling, Goethe University FrankfurtFrankfurt am Main, Germany

**Keywords:** multiple sclerosis, ceramides, sphinganines, sphingolipids, interferon-beta, fingolimod, natalizumab

## Abstract

Fingolimod is used for the treatment of multiple sclerosis (MS) and targets receptors for the bioactive sphingolipid sphingosine-1-phosphate (S1P). Whether fingolimod or other MS therapies conversely affect plasma concentrations of sphingolipids has, however, not yet been analyzed. Herein, we quantified 15 representative sphingolipid species by mass spectrometry in plasma from relapsing-remitting MS patients currently under fingolimod (*n* = 24), natalizumab (*n* = 16), or IFN-β (*n* = 18) treatment. Healthy controls (*n* = 21) and untreated MS patients (*n* = 11) served as control groups. IFN-ß treatment strongly increased plasma level of C_16:0_, C_18:0_, C_20:0_, and C_24:1_ ceramides compared to healthy controls, untreated patients, or patients receiving fingolimod or natalizumab medication. Natalizumab treatment increased plasma concentrations of both S1P and sphinganine-1-phosphate, whereas fingolimod treatment did not affect any of these lipids. Correlations of sphingolipids with the Expanded Disability Status Scale and other disease specific parameters revealed no systemic change of sphingolipids in MS, independent of the respective treatment regime. These results indicate type I interferon treatment to cause a strong and specific increase in ceramide level. If confirmed in larger cohorts, these data have implications for the efficacy and adverse effects of IFN-β. Moreover, quantification of ceramides soon after therapy initiation may help to identify therapy-responsive patients.

## Introduction

MS is an autoimmune disorder characterized by the destruction of the myelin sheath by auto-reactive immune cells. It is the most common cause for permanent disability in young adults, with an estimated 2.5 million patients worldwide (Brinkmann et al., 2010). Eighty-five percent of MS patients are affected by the RRMS, characterized by isolated relapses followed by complete or incomplete recovery and episodes of relative clinical stability until the next relapse. According to a recently suggested mechanism of relapse induction, myelin-specific memory T cells may reside in lymphoid tissues of the lung, moving from lung-draining lymph nodes to the CNS in a bystander activation process during respiratory infections (Odoardi et al., 2012). At the BBB, T cells slow down, e.g., via interaction of α4-integrins with the vascular cell adhesion molecule VCAM-1, and penetrate the CNS with the help of lytic enzymes, e.g., the MMPs 2 and 9 (Agrawal et al., 2006). Here, T cells are activated by local myelin-presenting perivascular dendritic cells or microglia, resulting in upregulation of costimulatory molecules (e.g., CD80) and the formation of chemokines and cytotoxic cytokines (Greter et al., 2005). During disease progression, and depending on the individual genetic disposition, myelin sheath damage develops into a multi-causal process involving activated B lymphocytes, antibody-dependent pathology, CD8^+^-driven direct cytotoxicity, autoinflammatory monokines, and reactive oxygen species (ROS)-dependent damage (Brinkmann et al., 2010; Halmer et al., 2014).

Sphingolipids, first identified in brain extracts in 1884, play a role in various diseases, and a major role in MS (Tudichum, 1884; Jana and Pahan, 2010). Ceramides are synthesized *de novo* by ceramide synthases in the endoplasmic reticulum. Here, four enzyme groups convert serine and palmitoyl-CoA to 3-ketosphinganine, and subsequently to sphinganine (also called dihydrosphingosine), which is converted in turn to dihydroceramide and ceramide (**Figure 3**). Subsequently, conjugation of a choline-phosphate group to ceramide leads to the formation of sphingomyelin, while conjugation of galactose to ceramide leads to the formation of galactosylceramide. With the help of ceramidases and sphingosine kinase 1/2, ceramides can be metabolized to sphingosine and subsequently to S1P. Besides *de novo* generation of ceramides, they can also be produced by “salvage” pathways, either by breaking down S1P to sphingosine and ceramide, or by recycling complex sphingolipids, (e.g., sphingomyelin) via the ASM (Don et al., 2014). Interestingly, ASM and the resulting ceramide release have been identified as a major mechanism of depression. Mice overexpressing ASM exhibit depression-like behavior even in the absence of stress, and antidepressants such as amitriptyline and fluoxetine mediate their therapeutic effects by inhibiting ASM activity. Furthermore, injection of C_16:0_ ceramide into the hippocampus is sufficient to induce depression-like behavior in WT mice (Gulbins et al., 2013). Downstream of ceramides, S1P is another very important signaling molecule, especially in MS. By activating five known S1PRs on the plasma membrane of various cells, S1P influences cellular processes such as the cell cycle, apoptosis and the regulation of cytokine expression (Schröder et al., 2011; Arlt et al., 2014; Ottenlinger et al., 2016). Furthermore, a steep S1P gradient between blood and secondary lymphoid compartments regulates the egress of lymphocytes out of secondary lymphoid organs.

The partial S1PR antagonist fingolimod (Gilenya^®^, Novartis, Basel, Switzerland; codenamed FTY720), approved by the FDA in 2010 as the first oral treatment for MS, has been shown to reduce the ARR by approximately 50% relative to placebo (Kappos et al., 2010). Its additional effects include activation of astrocytic or neuronal S1PRs by FTY720-P or the inhibition of IFN-γ formation by non-phosphorylated FTY720 (Groves et al., 2013; Ottenlinger et al., 2016). The humanized anti-α4-integrin antibody natalizumab (Tysabri^®^, Biogen, Cambridge, MA, USA) blocks the migration of T cells across intracerebral vessel walls, resulting in an ARR of 68% (Polman et al., 2006). However, long-term Tysabri-treated patients displayed an increased risk of progressive multifocal leukoencephalopathy (PML), an opportunistic viral infection of the brain which can lead to severe disability or death. Therefore, Tysabri-treated patients are regularly checked for anti-John Cunningham virus (JCV) antibodies (Sadiq et al., 2010). IFN-β (e.g., Avonex^®^, Biogen, Cambridge, MA, USA) also reduces the relapse rate, reaching an ARR of approximately 34% (The IFNB Multiple Sclerosis Study Group, 1993). MS patients reveal lower levels of circulating type I IFN than healthy controls and it is therefore believed that treatment with recombinant IFN-β suppresses disease progression (Feng et al., 2012). Pharmacodynamically, IFN-β dampens T cell proliferation and migration, reduces IFN-γ-release, diminishes IFN-γ mediated MHC class II expression, inhibits MMPs, increases IL-10 release, and co-activates regulatory T cells (Weinstock-Guttman et al., 2008). Side effects include flu-like symptoms and injection site complications (Walther and Hohlfeld, 1999). However, due to a discrepancy between the physiological and therapeutic effects of IFN-β treatment, its actual mode of action remains unclear.

Treatment of patients with MS is still challenging for neurologists: Although an increasing range of drug options is now available, it is not possible to identify therapy non-responders until they suffer further relapse and disability progression. Moreover, most MS drugs are associated with severe side-effects which often necessitate switches of medication. There is therefore a strong need for biomarkers which can guide treatment choices by distinguishing responders from non-responders. Notably, fingolimod specifically targets receptors for the bioactive sphingolipid S1P, but whether fingolimod or other MS medications conversely affect plasma sphingolipid concentrations has yet to be determined. Therefore, we quantified 15 representative sphingolipid metabolites by mass spectrometry in the plasma of relapsing-remitting MS patients currently treated with fingolimod, natalizumab, or IFN-β.

## Materials and Methods

### Patient Selection

Plasma samples of 69 RRMS patients were obtained from the biobank of the local biobanking project of the Department of Neurology at the University Clinic Frankfurt am Main, Germany. These had been drawn during the routine neurological diagnostic and checkup visits between 2011 and 2015 and stored at -80°C until further preparation. Plasma samples from 21 controls were obtained from healthy blood donors at the blood donation center in Frankfurt am Main (Blutspendedienst Hessen) in 2015. These samples were drawn during blood donation and were prepared and stored likewise. The study was performed in accordance with the Declaration of Helsinki and approved by the local ethics committee (reference number #110-11 for the biobank and #429/14 for sample analysis). All participants gave written informed consent prior to study inclusion. Inclusion criteria were diagnosis of RRMS, treatment with fingolimod or IFN-β (Rebif^®^, Avonex^®^, or Extavia^®^) for more than 3 months or natalizumab for more than 6 months, and an age of 18 – 60 years. Exclusion criteria comprised other disease-modifying treatments, other immunomodulatory treatments and other forms of MS. All patients were diagnosed by specialists in neurology. The EDSS score was not routinely evaluated at every checkup visit and was therefore not available for all patients. Blood from MS patients in relapse was taken before treatment with cortisone or other relapse-specific therapies.

### Determination of Sphingolipid Concentrations by High-Performance Liquid Chromatography Tandem Mass Spectrometry

Quantification of plasma sphingolipids was performed by high-performance liquid chromatography tandem mass spectrometry. For quantification of sphingolipids, 20 μl plasma was extracted twice with methanol:chloroform:HCl (15:83:2, v/v/v). The collected organic phases were evaporated at 45°C under a gentle stream of nitrogen and reconstituted in 50 μl methanol. Thereafter, liquid chromatography coupled to tandem mass spectrometry (LC-MS/MS) was used to assess quantities of C_14:0_ C_16:0_, C_18:1_, C_18:0_, C_20:0_, C_24:1_, C_24:0_ ceramide, C_16:0_, C_18:0_, C_24:0_, C_24:1_ sphinganine and the internal standard C_17:0_ ceramide, in addition to sphingosine, sphingosine1-phosphate, sphinganine and sphinganine1-phosphate and the internal standards (sphingosine-D7, sphinganine-D7, and sphingosine1-phosphate-D7). A Luna C18 column (150 mm × 2 mm ID, 5 μm particle size, 100 Å pore size; Phenomenex, Aschaffenburg, Germany) was used for chromatographic separation. The HPLC mobile phases consisted of water-formic acid (100:0.1, v/v) (A) and acetonitrile–tetrahydrofuran–formic acid (50:50:0.1, v/v/v) (B). For separation, a gradient program was used at a flow rate of 0.3 ml/min. The initial buffer composition 60% (A)/40% (B) was held for 0.6 min and then in 3.9 min linearly changed to 0% (A)/100% (B) and held for 6.5 min. Subsequently, the composition was linearly changed within 0.5 min to 60% (A)/40% (B) and then held for another 4.5 min. The running time for every sample (injection volume: 15 μl for determination of ceramides and sphinganines and 10 μl for the other sphingolipids) was 16 min. MS/MS analyses were performed on a API4000 (triple quadrupole mass spectrometer) equipped with an APCI (Atmospheric Pressure Chemical Ionization) ion source (Sciex, Darmstadt, Germany) for determination of ceramides and sphinganines, and with an ESI (Electrospray Ionization) ion source for determination of sphingosine, sphinganine, and their 1-phosphate derivatives. The analysis was done in MRM mode. For every analyte, two transitions were recorded: one for quantification and a second for qualification, to exclude false positive results, with a dwell time of 50 ms. For analysis and quantification, the Analyst Software 1.6 (Sciex, Darmstadt, Germany) was used and the peak area of each analyte was corrected by the peak area of the corresponding internal standard. Linearity of the calibration curve was proven for C_16:0_, C_24:1_, C_24:0_ ceramide; C_16:0_, C_24:1_, and C_24:0_ sphinganine from 12 to 3000 ng/mL, for C_18:0_, C_18:1_ ceramide from 1.2 to 300 ng/mL, for C_20:0_ ceramide, C_18:0_ sphinganine from 5 to 500 ng/mL and for C_14:0_ ceramide from 4 to 100 ng/mL. For sphingosine, sphinganine, and their phosphate derivatives, the calibration curve ranged from 0.25 to 250 ng/mL. The coefficient of correlation was at least 0.99. Variations in accuracy were less than 15% over the whole range of calibration, except for the lowest limit of quantification, where a variation in accuracy of 20% was accepted.

### Statistical Analysis

Statistical analysis was done using Graph Pad Prism 5 (La Jolla, CA, USA) and SPSS 20 (Chicago, IL, USA). Normal distribution was assessed using a Kolmogorov–Smirnov test. Normally distributed parameters were analyzed with two-tailed *t*-tests or One-way ANOVAs with Tukey’s *post hoc* comparison. Non-normally distributed parameters were analyzed using Mann–Whitney’s U and Kruskal–Wallis’ tests with Dunn’s *post hoc* comparison. Correlations were analyzed using Spearman or Pearson correlation coefficients, respectively. Sphingolipid concentrations are shown as Tukey box plots and statistical significant events are indicated with, ns‘*p* > 0.05, ^∗^*p* ≤ 0.05, ^∗∗^*p* < 0.01, and ^∗∗∗^*p* < 0.001.

## Results

The analysis of 15 representative sphingolipids was performed in plasma from 69 differently medicated RRMS patients and 21 matched healthy controls (**Table 1**). Of the 69 MS patients, 16 were treated with natalizumab, 24 with fingolimod, 18 with IFN-β and 11 were untreated at that time. While some patients were in remission, a high number of relapsing patients were enrolled into the study to enable further analysis of the effect of relapse on plasma sphingolipids. In relapsing patients, blood samples were taken before cortisone therapy was initiated. Of the 15 analytes, C_18:1_ ceramide, sphingosine and sphinganine did not fulfill the quality control criteria (CV < 20%), because their concentrations were only just above the detection limit (data not shown). These analytes were therefore excluded from further analyses, leaving a total of 12 analytes which were further analyzed. Samples from RRMS patients did not reveal a correlation with storage time (Pearson or Spearman correlation coefficient: -0.29 ≤*R* ≤ 0.16, data not shown). Additionally, there was no correlation of sphingolipids with age (Pearson or Spearman correlation coefficient: -0,147 ≤*R* ≤ 0,257, data not shown) and no major influence of gender (data not shown). Concerning the latter, C_14:0_ ceramide was increased exclusively in female RRMS patients (Mann–Whitney’s *U* test, *p* = 0.017), but this was not seen in healthy controls (Mann–Whitney’s *U* test, *p* = 0.353). Plasma sphingolipid levels in RRMS patients in remission versus patients in relapse showed no notable difference (**Table 2**). Exclusively C_18:0_ ceramide was increased in RRMS patients in relapse treated with IFN-β, but this was not seen in other treatment regimes. Therefore, in the subsequent analysis, patients in remission were analyzed together with those in relapse. When sphinganines and ceramides from MS patients were compared to healthy controls, a tendency was observed for all ceramides to be increased in MS patients. However, this increase reached significance only for C_24:1_ ceramide and C_16:0_ sphinganine (**Table 3**). dhS1P was elevated in MS patients compared to healthy controls. But, these increases were rather associated with the individual treatment regimens than with the disease itself (see below).

**Table 1:** Demographic details of RRMS patients and healthy controls.

Treatment group	Healthy controls	w/o	IFN-β	Natalizumab	Fingolimod
*n*	21	11	18	16	24
Sex [w/m]	15/6	7/4	16/2	11/5	17/7
Age [mean]	40.4	37.5	39.9	36.5	39.2
Age [range]	22.1–55.6	23.5–53.8	23.3–59.36	23.9–53.2	26.0–53.08
In relapse/remission [*n*]	n/a	11/0	5/13	7/9	8/16
Disease duration [years, mean]	n/a	6.0	7.2	7.2	11.9
Disease duration [years, range]	n/a	0–14.7	0.1–18.7	1.2–14.4	1.69–32.0
EDSS [mean, *n*]	n/a	1.5, *n* = 2	2.9, *n* = 7	2.8, *n* = 8	3.8, *n* = 16
EDSS [range]	n/a	1–2	2–4	1–7.5	2–6.5

*Disease duration of 0 indicates blood sampling at initial diagnosis. Missing data: The EDSS score was not evaluated on the date of sample preparation in all cases. n/a, not available, w/o, RRMS patients without therapy.*

**Table 2:** RRMS patients in remission and in relapse reveal no obvious differences in plasma sphingolipid concentrations.

Treatment group	IFN-β	Natalizumab	Fingolimod	All (with untreated)
Relapse/remission [*n*]	5/13	9/7	16/8	31/38
C_14:0_ ceramide	0.059	0.758	0.076	0.135
C_16:0_ ceramide	0.703	0.536	0.052	0.101
C_18:0_ ceramide	**0.035**	0.758	0.569	0.763
C_20:0_ ceramide	0.143	0.918	0.466	0.534
C_24:0_ ceramide	0.145	0.719	0.654	0.321
C_24:1_ Ceramide	0.479	0.937	0.475	0.766
C_16:0_ sphinganine	0.775	0.351	0.528	0.247
C_18:0_ sphinganine	0.336	1.000	0.653	0.914
C_24:0_ sphinganine	0.387	0.607	0.697	0.563
C_24:1_ sphinganine	0.173	0.681	0.417	0.866
S1P	0.913	0.958	0.580	0.719
dhS1P	0.633	0.252	0.610	0.971

*Patients in remission were compared to patients in a current relapse depending on their treatment regime. Data are shown as *p*-values from Mann–Whitney’s *U* or two-tailed *t*-tests and significant *p*-values (*p* ≤ 0.05) are highlighted in bold. dhS1P, sphinganine-1-phosphate; S1P, sphingosine-1-phosphate.*

**Table 3:** RRMS patients reveal a tendency of increased plasma ceramides compared to healthy controls.

	RRMS patients [*n* = 69]			Healthy controls [*n* = 21]			*p*-value
	mean	*SD*	median	mean	*SD*	median	
C_14:0_ ceramide	18.9	8.1	17.3	15.5	7.1	14.0	0.111
C_16:0_ ceramide	650.9	198.0	635.1	573.2	152.2	550.4	0.128
C_18:0_ ceramide	164.6	73.8	152.0	138.6	44.8	126.7	0.177
C_20:0_ ceramide	385.1	161.3	370.7	335.1	104.7	323.6	0.227
C_24:0_ ceramide	6936.6	2165.2	6707.6	6143.7	1695.6	6178.5	0.128
C_24:1_ ceramide	1560.4	496.5	1513.6	1187.6	452.2	1129.5	**0.003**
C_16:0_ sphinganine	31.5	15.4	29.3	24.1	12.6	24.5	**0.042**
C_18:0_ sphinganine	37.3	28.0	32.6	26.9	13.7	23.0	0.143
C_24:0_ sphinganine	368.7	172.6	335.1	307.9	118.5	268.7	0.100
C_24:1_ sphinganine	249.6	111.4	222.3	206.5	80.3	181.2	0.141
S1P	312.3	111.6	298.8	347.8	78.1	335.4	0.178
dhS1P	57.2	27.5	50.2	40.1	13.2	39.4	**0.006**

*Data are shown as mean, *SD*, and median with *p*-values of Mann–Whitney’s *U* or two-tailed *t*-tests with significant *p*-values (*p* ≤ 0.05) highlighted in bold. dhS1P, sphinganine-1-phosphate; RRMS, relapsing-remitting multiple sclerosis; S1P, sphingosine-1-phosphate.*

### IFN-β Treatment Specifically Increased Ceramide Concentrations in Plasma of RRMS Patients

**FIGURE 1:**
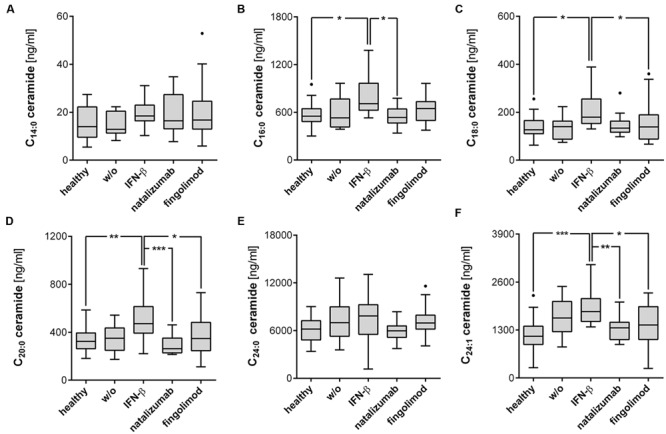
**IFN-β-treated RRMS patients demonstrated increased ceramides of specific chain length. (A–F)** Ceramides were determined by mass spectrometry from plasma of RRMS patients and healthy controls. Data shown are Tukey box plots with a horizontal line representing the median, whiskers representing the 1.5× interquartile range and dots representing outliers. Healthy controls (*n* = 21), untreated patients (w/o, *n* = 11), IFN-β (*n* = 18), natalizumab (*n* = 16), and fingolimod (*n* = 24) treated patients. One-Way ANOVA with Tukey’s *post hoc* comparison or Kruskal-Wallis’ test with Dunn’s *post hoc* comparison for normally or non-normally distributed data, respectively. ^∗^*p* ≤ 0.05; ^∗∗^*p* < 0.01; ^∗∗∗^*p* < 0.001.

**FIGURE 2:**
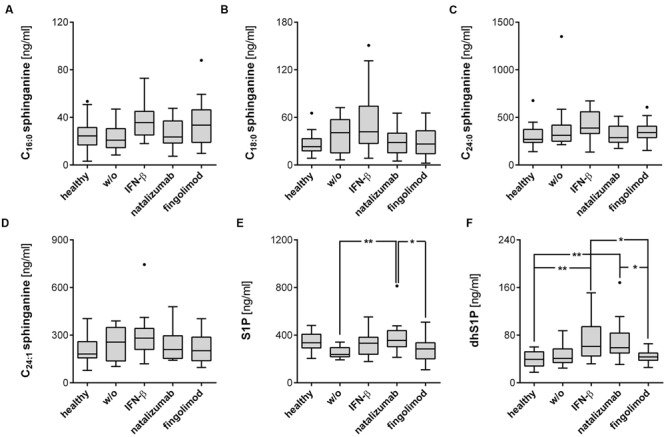
**S1P and dhS1P level are affected by the respective treatment regime. (A–D)** Sphinganines were determined from plasma of RRMS patients and healthy controls, as well as **(E,F)** S1P and dhS1P. Data shown are Tukey box plots with a horizontal line representing the median, whiskers representing the 1.5× interquartile range and dots representing outliers. Healthy controls (*n* = 21), untreated patients (w/o, *n* = 11), IFN-β (*n* = 18), natalizumab (*n* = 16), and fingolimod (*n* = 24) treated patients. One-Way ANOVA with Tukey’s *post hoc* comparison test or Kruskal-Wallis’ test with Dunn’s post hoc comparison for normally or non-normally distributed data, respectively. ^∗^*p* ≤ 0.05; ^∗∗^*p* < 0.01.

To a large degree, the increase in ceramides and sphinganines was not due to MS in general, but related to treatment, especially IFN-β therapy. IFN-β-treated patients revealed increased C_16:0_, C_18:0_, C_20:0_, and C_24:1_ ceramides compared to healthy controls (**Figures 1B–D,F**). These ceramides were also elevated compared to patients receiving other treatment regimens or no treatment, but did not reach statistical significance in all cases. C_14:0_ ceramide and C_24:0_ ceramide were unaffected by the treatment (**Figures 1A,E**). C_18:1_ ceramide did not pass the quality control, but was also elevated in IFN-β-treated patients compared to healthy controls (*p* < 0.01) or natalizumab-treated patients (*p* < 0.05, data not shown). Concerning sphinganine species, especially C_16:0_ and C_18:0_ sphinganine showed a tendency to be increased in IFN-β-treated patients compared to healthy controls, but this did not reach statistical significance (**Figures 2A,B**, *p* = 0.06). S1P was slightly decreased in untreated patients compared to healthy controls (*p* = 0.095) and especially natalizumab treatment increased S1P on a level comparable to healthy controls (**Figure 2F**). Interestingly, patients treated with the S1PR antagonist fingolimod exhibited no statistically significant difference of S1P levels compared to the control group. Furthermore, patients receiving natalizumab or IFN-β showed increased dhS1P concentrations compared to healthy controls (**Figure 2G**).

### Ceramide and Sphinganines are not Affected by the Disease Status of RRMS Patients

Since IFN-β treatment strongly affected ceramide and sphinganine levels, further analysis was required to assess whether sphingolipid concentrations are affected by RRMS independently of the treatment regime. If it can be ruled out that the disease itself exerts an effect on sphingolipids, a specific increase of ceramide species may have potential as a marker of therapy response. Since IFN-β-treated patients were found to have elevated ceramide and sphingosine levels, they were omitted from the subsequent analysis (reduction to *n* = 56), except for the unaffected analytes C_14:0_ and C_24:0_ ceramide, and C_24:0_ and C_24:1_ sphinganine. Ceramides and sphinganines were assessed in relation to disease duration, time from sampling to the last or next relapse, and the EDSS score (**Table 4**). Only minor correlations with the disease duration were observed, indicating changes in analyte levels to be predominantly induced by treatment and not by the disease *per se*.

**Table 4:** Plasma sphingosines and ceramides are not affected by RRMS, independent from the treatment.

Correlation with	Disease duration	Time to last relapse	Time to next relapse	EDSS
*n* = (IFN-β/non-IFN-β)	69/56	67/54	29/27	33/27
C_14:0_ ceramide^a^	0.246^∗^	0.001	0.107	0.243
C_16:0_ ceramide^b^	0.152	-0.115	0.149	0.030
C_18:0_ ceramide^b^	0.006	-0.174	0.062	0.014
C_20:0_ ceramide^b^	0.101	-0.064	0.250	0.146
C_24:0_ ceramide^a^	0.296^∗^	0.112	0.022	0.112
C_24:1_ ceramide^b^	0.081	-0.254	0.078	0.218
C_16:0_ sphinganine^b^	0.237	0.057	0.201	-0.162
C_18:0_ sphinganine^b^	-0.092	0.008	0.052	-0.331
C_24:0_ sphinganine^a^	0.089	0.125	0.171	-0.105
C_24:1_ sphinganine^a^	-0.173	-0.049	-0.038	-0.197

*Disease duration, time to the last or the next relapse and EDSS score were correlated to ceramides and sphinganines using Pearson or Spearman correlations, respectively. Data are shown as the correlation coefficient (*R*) with ^∗^*p* ≤ 0.05. Missing data: since IFN-β therapy affected specific plasma ceramides and sphinganines, especially for these analytes patients treated with IFN-β were omitted; a = including patients treated with IFN-β; b = without patients treated with IFN-β; time to last relapse = two patients had their first relapse and were excluded; time to next relapse = only patients with a relapse before data collection was begun were included; EDSS = only patients with EDSS score evaluation on date of sample collection were included.*

## Discussion

The oral prodrug fingolimod is known to act on the receptors for the bioactive sphingolipid S1P in secondary lymphoid organs. To date, no studies have examined a possible inverse effect of fingolimod and other MS drugs on the metabolism of sphingolipids. Our results indicate that no such effect is associated with fingolimod, but did reveal a strong and specific increase of ceramides of specific chain lengths especially in IFN-β-treated MS patients, compared to healthy controls, untreated patients or other treatment groups (**Figures 1** and **2**).

### The Therapeutic Effect of IFN-β and Associated Side-Effects

IFN-β is believed to support regulatory functions of the immune system in MS, but the actual mode of action is not completely understood. Major side-effects of IFN-β therapy include injection site reactions and flu-like symptoms. Furthermore, IFN-β has been suggested to cause or exacerbate depression. Whereas this observation failed to reach significance on a single trial level, pooled data from several clinical trials clearly showed that IFN-β increased the rate of depression from 8% in the placebo-treated group to 5–18% in patients treated with 22–44 μg IFN-β via different administration routes (*p* = 0.017) (Patten et al., 2005). Accordingly, a switch from an injectable disease-modifying therapy (IFN-β or glatiramer acetate) to oral fingolimod improves depressive symptoms in patients with RRMS (Hunter et al., 2016).

### Analogous to IFN-β-Treated MS Patients, Patients with Depression, Systemic Lupus Erythematosus or Parkinson’s Disease are Characterized by Increased Plasma Ceramides

**FIGURE 3:**
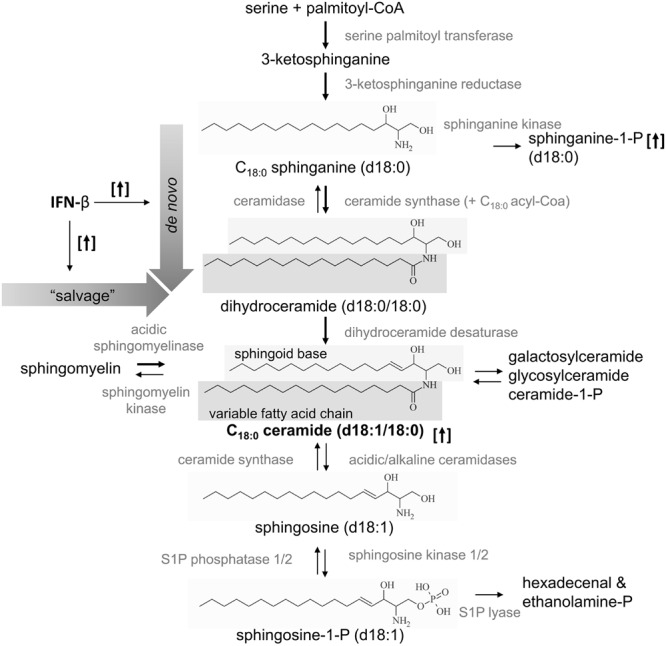
**Schematic overview of the generation of C_18:0_ ceramide and the supposed effect of IFN-β.** Ceramides are produced *de novo* by serine and palmitoyl-CoA or by “salvage” pathways, with recycling of sphingomyelin or sphingosine-1-phosphate. IFN-β increased e.g., C_18:0_ ceramide and sphinganine-1-phosphate (with a C_18_-backbone) as indicated by the arrows in brackets. Accordingly, the *de novo* synthesis and/or the “salvage” pathway may be activated by IFN-β therapy as indicated by bold arrows.

Our data reveals that IFN-β specifically increased plasma ceramide level, especially C_16:0_, C_18:0_, C_20:0_, and C_24:1_ species, compared to healthy controls, untreated patients or other treatment groups (**Figure 1**). C_16:0_ and C_18:0_ sphinganine showed a tendency to be increased compared to healthy controls (**Figures 2A,B**, *p* = 0.06). S1P was not affected by IFN-β-treatment, but dhS1P was increased compared to healthy controls (*p* < 0.01, **Figure 2F**). Diverse pro-inflammatory cytokines, such as IFN-α, TNF-α, IL-1β, or IFN-γ induce sphingolipid metabolizing enzymes (Jenkins et al., 2010; Su et al., 2011) and especially IFN-α has already been shown to effect a decrease, for example, in HDL cholesterol in hepatitis C patients treated with IFN-α (Shinohara et al., 1997). Accordingly, a direct influence either of IFN-β itself or of IFN-β-induced cytokines on the metabolism of sphingolipids is to be expected. As mentioned above, ceramides can either be produced by the *de novo* pathway with the help of ceramide synthases or, alternatively, via “salvage” pathways, e.g., by recycling of sphingomyelin by the ASM or by recycling of S1P by the S1P phosphatases 1/2 (**Figure 3**). Focusing on the “salvage” pathway originating from sphingomyelin, ASM is a ubiquitously expressed enzyme, activated by a variety of stress stimuli, e.g., IFN-α, TNF-α, IL-1β, or IFN-γ (Jenkins et al., 2010; Su et al., 2011*)*. Non-MS patients with severe major depression have been found to have increased ASM activity in peripheral blood mononuclear cells (Kornhuber et al., 2005). Similarly, non-MS study participants with recent major depression within the previous 2 years reveal increased plasma levels of C_16:0_, C_18:0_, C_20:0_, C_24:1_, and C_26:1_ ceramide compared to subjects with less recent prior depression (>2 years before) or no history of depression. C_22:0_, C_24:0_, and C_26:0_ were not affected (Gracia-Garcia et al., 2011). These ceramide species are identical to the ones affected by IFN-β in our cohort.

While IFN-β reveals therapeutic effects in MS, increased type I IFN signaling is associated with SLE (Crow, 2010). Checa et al. detected increased concentrations of certain sphingolipids in SLE patients’ plasma compared to healthy controls, namely C_16:0_, C_18:0_, C_20:0_, and C_24:1_ ceramide, while C_14:0_, C_22:0_, and C_24:0_ were unaffected (Checa et al., in preparation, preliminary results with a lower number of patients published (Checa et al., 2016)). This characteristic pattern of ceramides resembles that observed in IFN-β-treated MS patients in our cohort (**Figures 1** and **2**) or individuals with recent major depression as reported by Gracia-Garcia et al. (2011). The same ceramides are also elevated in patients suffering from sporadic Parkinson’s disease with cognitive impairment (Mielke et al., 2013). Here again, C_16:0_, C_18:0_, C_20:0_, C_22:0_, C_24:1_, and C_26:1_ were elevated compared to patients without cognitive impairment, whereas C_22:1_, C_24:0_, and C_26:0_ ceramides were not affected. The same ceramide species were elevated compared to healthy controls albeit with slight differences in their level of significance. In summary, IFN-β treatment of RRMS specifically increased ceramides of certain specific chain-lengths. These ceramides are also elevated in patients with depression, SLE or sporadic Parkinson’s disease.

### No Evidence for a Treatment-Independent Systemic Change of Sphingolipids in RRMS

To evaluate treatment-independent effects in RRMS, the influence of relapse, EDSS score, disease duration and time to the previous and next relapse were analyzed (**Tables 2** and **4**). It is important to note that not only S1P but also a number of other sphingolipids play an important role in the pathophysiology of MS. About 80% of the myelin sheath consists of lipids, predominantly sphingomyelins and galactosylceramides (also called “cerebrosides”). Autoantibodies against these lipids are often found in patients with MS (Menge et al., 2005). C_18:0_ ceramide accumulates in human MS lesions, while C_16:0_, C_18:0_, and C_20:0_ ceramides were found in lesions in a cuprizone animal model of demyelination (Kim et al., 2012). On contact with neurons, the CSF of MS patients induces bioenergetic dysfunction and oxidative damage, due to increased C_16:0_ and C_24:0_ ceramides (Vidaurre et al., 2014). The major cellular source of ceramides in MS is reactive astrocytes, which show enhanced expression of enzymes involved in sphingolipid metabolism (van Doorn et al., 2012). Interestingly, levels of the hexosylceramide HexCer_16:0_ in CSF correlated with the EDSS score of MS patients, indicating an accumulation over the course of disease (Checa et al., 2015). In neurodegeneration, especially in Alzheimer’s disease, ceramides have been shown to be increased in CSF and most brain regions (Mielke and Lyketsos, 2010). Furthermore, high plasma ceramides have been associated with greater disease progression (Mielke et al., 2012). In RRMS, however, we found no evidence of a treatment-independent systemic change in sphingolipids. Although ceramides have been shown to be locally released in MS lesions, increases in plasma ceramides comparable to those seen in patients with Alzheimer’s disease were not observed. Furthermore, especially untreated RRMS patients failed to show statistically significant differences compared to healthy controls. Therefore, ceramides and sphinganines are not influenced by the disease *per se*, but are increased due to specific effects of IFN-β therapy.

In summary, we identified for the first time a previously unknown effect of IFN-β treatment on plasma ceramides: We found elevated levels of C_16:0_, C_18:0_, C_20:0_, and C_24:1_ ceramides in MS patients receiving IFN-β. The very same ceramides have already been shown to be elevated in patients with depression, SLE or sporadic Parkinson’s disease, thus indicating a molecular connection. As a prerequisite for biomarker development, we were able to demonstrate that ceramide and sphinganine levels are not affected by RRMS *per se*. Further research will be necessary to discover whether ceramide induction by IFN-β occurs as a result of increased ASM activity or due to the induction of other enzymes. In addition, further studies are required to assess the utility of ceramide induction as a biomarker in therapy responders, and to discover whether ceramide induction is involved in side-effects of IFN-β therapy.

## Author Contributions

FO analyzed all data, wrote the manuscript, performed statistics, and designed the figures. CM helped designing the study, performed and supervised sample collection, and recruitment of patients and corrected the manuscript. NF and YS performed the LC-MS/MS determination of sphingolipids. AS and KS helped by designing the study, data interpretation, and writing the manuscript. HA helped with statistics and data analysis. JP supplied basic lab equipment. HR had the idea, designed and closely supervised all experiments, checked all data in detail and finalized the manuscript.

## Conflict of Interest Statement

CM received travel grants from Genzyme pharma. All the other authors declare that the research was conducted in the absence of any commercial or financial relationships that could be construed as a potential conflict of interest.

## Abbreviations

ARR: annualized relapse rate
ASM: acidic sphingomyelinase
BBB: blood brain barrier
CNS: central nervous system
CSF: cerebrospinal fluid
CV: coefficient of variation
dhS1P: sphinganine-1-phosphate
EDSS: Expanded Disability Status Scale
IFN: Interferon
MMPs: matrix metalloproteinases
MS: multiple sclerosis
n/a: not available
RRMS: relapsing-remitting multiple sclerosis
S1P: sphingosine-1-phosphate
S1PR: sphingosine-1-phosphate receptor
SLE: systemic lupus erythematosus
w/o: without
